# A double-crystal bent Laue parallel-beam compressor

**DOI:** 10.1107/S1600577525009609

**Published:** 2026-01-20

**Authors:** Glendon Rhoades, L. Dean Chapman

**Affiliations:** a Department of Anatomy, Physiology and Pharmacology University of Saskatchewan Saskatoon SK CanadaS7N 5A2; RIKEN SPring-8 Center, Japan

**Keywords:** beam compressor, bent Laue diffraction, double-crystal monochromator, biomedical imaging, polychromatic beam, spectral CT, computed tomography, plant imaging

## Abstract

We demonstrate the ability to manipulate a synchrotron X-ray beam’s size, maintaining a nearly parallel beam, by ‘compressing’ it horizontally using a pair of cylindrically bent Laue crystals. This compressed beam creates a desirable aspect ratio for computed tomography (CT) of small samples. In contrast to collimation of the beam, this compressor ‘funnels’ a large horizontal section of the beam’s flux into a smaller imaging area, raising intensity and allowing users to work in the gap between bend-magnet (BM) and insertion-device beamline intensities. We demonstrate here the proof-of-principle of this optical system with CT of multiple small samples. This system can increase the capability of CT at synchrotron facilities by allowing more flexibility in the intensity of BM beamlines. It also produces a spectral beam which will be further explored in future papers.

## Abstract 2

An application of bent Laue crystals as X-ray optics to increase beam intensity in bending-magnet beamlines at synchrotron facilities is presented.

## 1. Introduction

Aspect ratios of 3 × 5, 8 × 10, 5 × 7 *etc*. are commonly used in imaging of all types because of their convenience and ability to capture meaningful information of most subjects. Moving towards aspect ratios of 10 × 1, 50 × 1, or even 100 × 1, the information captured by the image becomes increasingly one-dimensional.

Bending-magnet beamlines inherently produce high-aspect-ratio beams. Line scan mode, often utilized at bending-magnet beamlines, treats the beam as one-dimensional and uses scanning to capture information in a second dimension. For many imaging techniques, line scan mode proves complicated or completely impractical due to sample movement, motion artifacts or incompatibility with imaging techniques [*e.g.* high-resolution computed tomography (CT)].

The real issue is that for large samples the beam is approaching the limitation of one-dimensional images, and for small samples most of the beam is unused. Creating an aspect ratio of the imaging beam nearer to 1:1 greatly improves the usefulness of the images produced. There are two efficient ways of achieving this, in theory. One is to expand the beam vertically, increasing the height dimension towards that of the width. The other is to compress the beam horizontally, decreasing the width dimension towards that of the height, which we present here. For the purposes of this paper ‘compression’ of the beam refers to reducing the beam’s spatial dimensions while attempting to maintain the number of X-rays previously occupying the larger beam area; this is done using crystal optics.

For the BioMedical Imaging and Therapy (BMIT) bending-magnet beamline at the Canadian Light Source, in the sample hutch the beam can be over 200 mm wide (Wysokinski *et al.*, 2007 ▸). The maximum horizontal photon beam angle is 19.54 mrad, of which 10 mrad is utilized. The aspect ratio for a beam of this geometry is greater than 50:1 (horizontal size: vertical size). However, the vast majority of experiments performed on the BMIT bend magnet use a beam width less than 100 mm (though maximum beam width is 200 mm), and many samples are less than 10 mm wide (Belev *et al.*, 2015 ▸).

Altering of the beam dimensions (other than by collimation) has been demonstrated previously using asymmetrically cut crystals and mirror optics. Asymmetrically cut crystal systems have been used to produce microbeams using a monolithic block of perfect silicon crystal (<10 µm beam dimensions) (Korytár *et al.*, 2005 ▸; Christensen *et al.*, 1992 ▸). Focusing by concave spherical mirrors at grazing incidence (Kirkpatrick & Baez, 1948 ▸) becomes somewhat impractical at higher energy (>20 keV) due to the length required to accept an appreciably sized beam. Multilayer coated mirrors can be used but also require increased length at higher X-ray energies. There are other focusing techniques that are used to produce a ‘point’ beam, but are not suitable for imaging applications.

Recent research (Martinson *et al.*, 2015 ▸) has made strides in improving beam dimensions for large samples. That technique involved using a pair of confocally bent Laue crystals to expand the vertical dimension of a bend-magnet (BM) beam and yielded a usable beam with dimensions of 75 mm (width) by 50 mm (height).

From a beamline optics perspective, the natural progression from the work of Martinson *et al.* was to approach the aspect-ratio problem from the opposite end: development of a compressor for the horizontal dimension of the beam, bringing the beam into a small area with a desirable aspect ratio. This approach gives a more intense beam (relative to a standard monochromatic BM beam) with desirable aspect ratios and dimensions for small-sample imaging. To our knowledge, this has not been demonstrated previously and forms the logical complement to the beam expander just described.

Crystals can be used to manipulate the beam’s properties in many ways. If the crystal is bent, the lattice planes will be curved, such that the angle of the diffracted beam, and thus the selected energy, changes across the bent crystal. It should be noted that the X-ray beam diffracted by a bent crystal is spectral in nature; this property is explored further in the *Discussion*. When coupled with another confocally bent crystal, the two optical elements can change the size of the beam while maintaining the parallel nature of the incoming beam (this is in addition to the spectral effect on the beam). It should be noted that several double bent Laue monochromators are currently being used primarily for high-power high X-ray energy applications. These devices use bent Laue crystals primarily to increase the bandwidth and thus flux, but do not appreciably affect the beam size.

**Figure 1:**
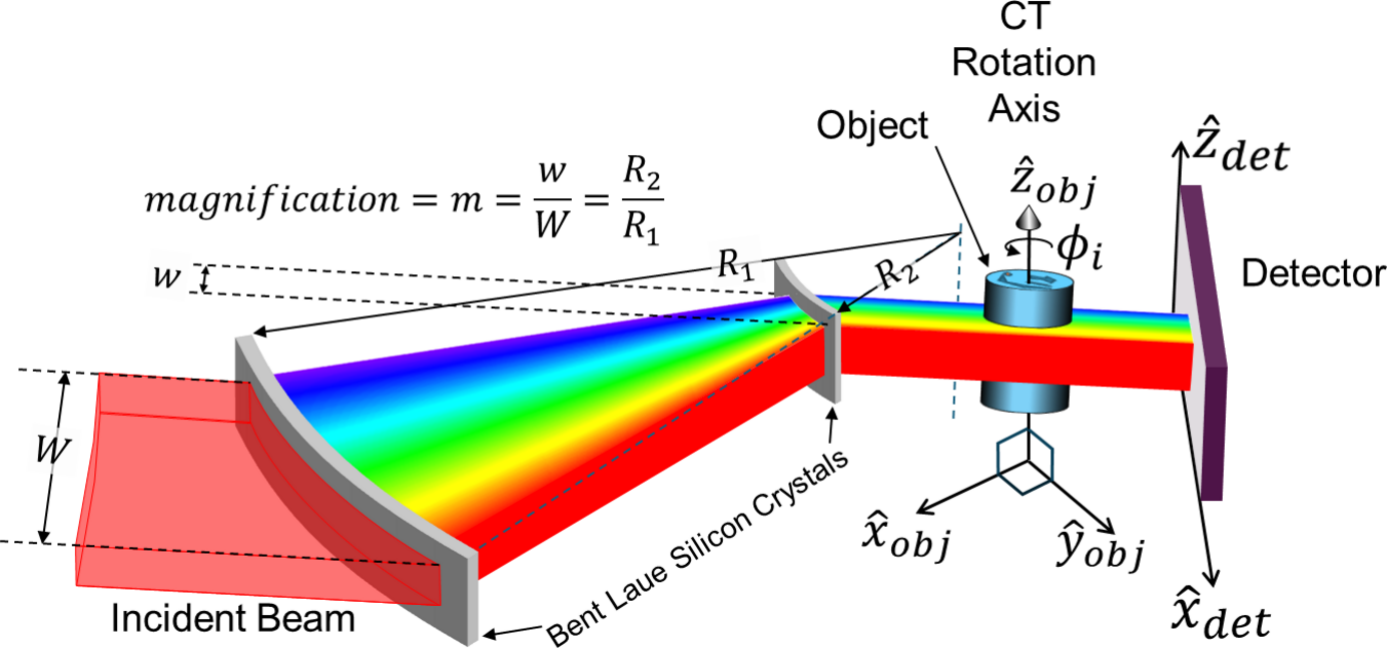
Perspective view of the parallel-beam compressor using a pair of bent Laue crystals.

Bending of a crystal plate in the Laue or transmission geometry results in a real or virtual focus from the overall bending of the lattice planes; this focus is referred to as the geometric focus (see Fig. 1 ▸). Also, as each incident ray penetrates the crystal the diffraction occurring along the beam path also creates a family of rays that focus (either real or virtually); this focus is referred to as the polychromatic or single ray focus. We have chosen to (as much as possible) match these two focal lengths to minimize beam blurring of the diffracted rays. This matching of the two foci is referred to as the ‘magic condition’ (Qi *et al.*, 2021 ▸; Schulze *et al.*, 1998 ▸). This condition places beams of the same energies at the same locations, regardless of whether they arise geometrically or polychromatically.

While this condition only occurs for a specific energy and is dependent on the lattice plane asymmetry angle, in our case, the photon energy that best meets the condition is near 30 keV and deviations in energy do not create significant blurring effects.

## 2. Materials and methods

**Figure 2:**
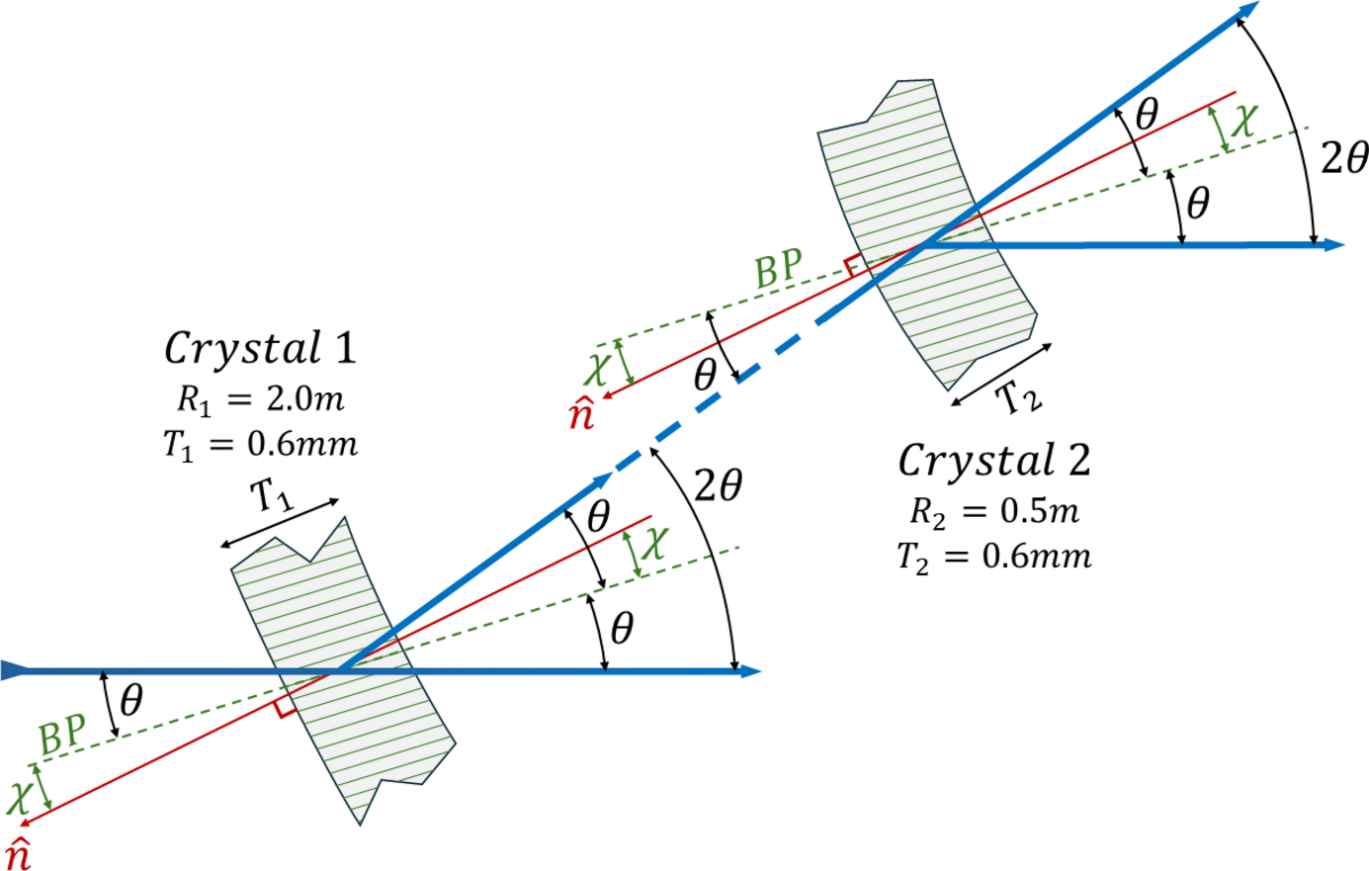
Schematic of the two-crystal compressor arrangement showing the approximate relationship between an X-ray beam (blue), the lattice planes (green) and the surface normal, 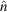 (red). The angles that the rays make relative to the crystal’s lattice planes (θ) and the lattice planes’ (BP) inclination relative to the crystal surface (χ) are indicated. A single ray is shown that interacts with the two crystals to indicate in more detail the diffraction geometry.

Feasibility experiments were performed at the Canadian Light Source, on the BMIT BM beamline. The crystals used were 0.6 mm-thick single-crystal silicon wafers with (5,1,1) surfaces. Both crystals had a 5-inch diameter, and an asymmetry angle of 3.33° for the (3,1,1) reflection type used. The incident angle of the X-rays to the crystal face is then set to the asymmetry angle plus the Bragg angle on the first crystal (see Fig. 2 ▸). The focusing geometry was used, such that the geometric and polychromatic foci of the two crystals matched, or nearly matched; this roughly satisfied the ‘magic condition’ as noted earlier.

**Figure 3:**
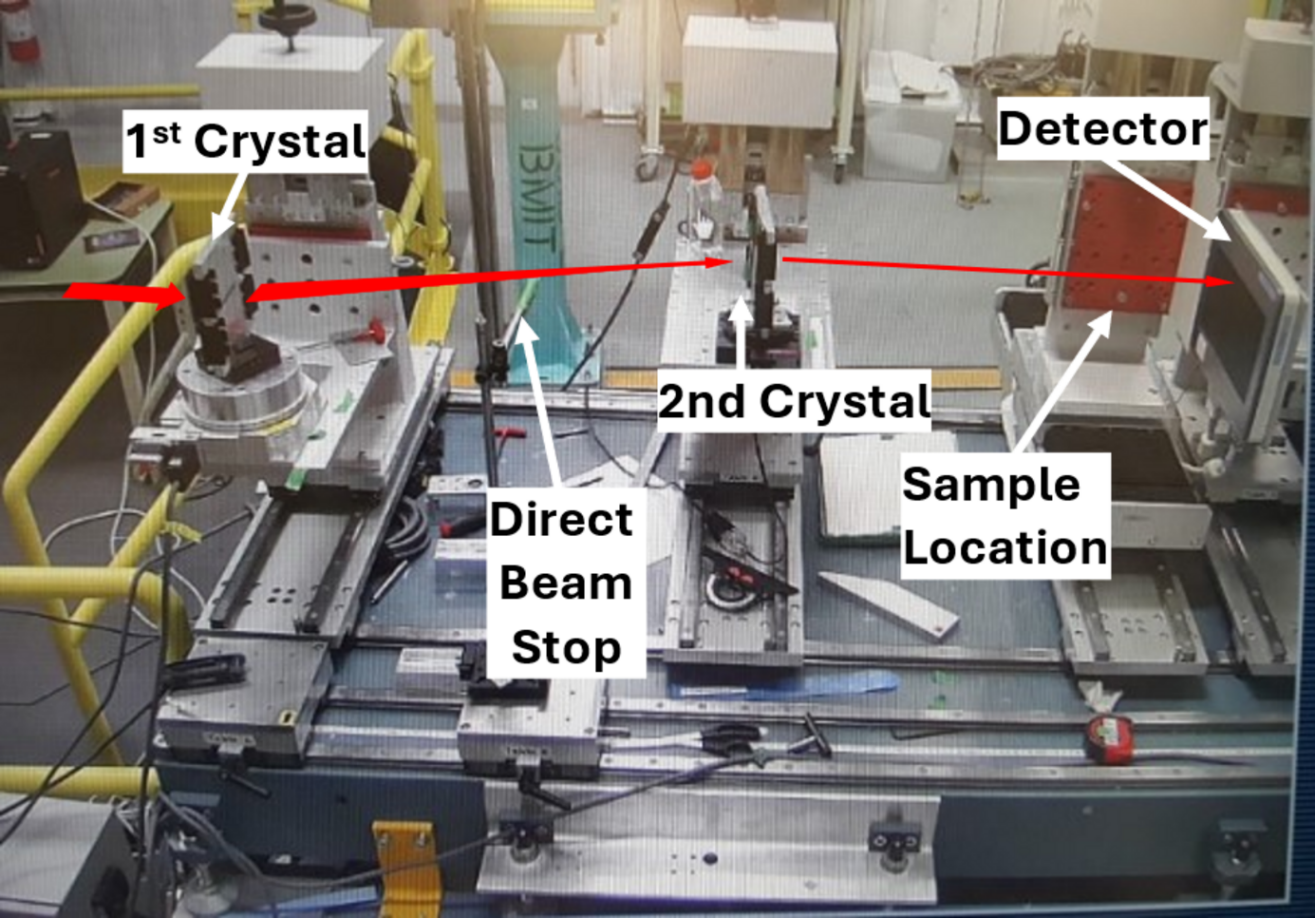
Setup of two mounted crystals, sample stage and detector at the beamline. The compressed beam path is shown in red. The distance along the incident beam path from the first crystal to the plane of the detector is approximately 135 cm. The vertical translation stage that holds the sample is shown, but not with the sample alignment and rotary stages.

The ratio between the two crystals’ bending radii gives the compression factor of the beam, as well as determining the distance between the two crystals relative to the focus (see Fig. 1 ▸). In our experiments, the first crystal is bent to a radius of 2.0 m, and the second crystal to a radius of 0.5 m, and arranged such that foci coincide, and the beam’s width is reduced by a factor of 4 (0.5 m/2 m), resulting in a beam width of approximately 22.5 mm of which 12 to 14 mm was used in these experiments. The crystals are mounted on curved aluminium blocks to maintain correct bending radius and the beam passes through a window machined into each of the blocks (see Fig. 3 ▸).

The first crystal is centered on the incident filtered white beam (1.1 mm Al, 0.5 mm Be, 61.7 mm water) and oriented such that the beam is diffracted outboard from the storage ring. The beam is collimated to the width of the window in the aluminium block and lead shielding is used to stop the transmitted beam through the crystal. An iodine solution is placed in the diffracted beam, and the crystal’s angle relative to the incoming beam is adjusted until the iodine *K*-edge is near the middle in the 3,1,1 reflected beam. The focus is then found using a fluorescent screen, and the second crystal is centered on the beam at 25% of the distance from the focus to the first crystal. The focus location was determined to be 91.3 cm in these experiments, so the second crystal was placed 68.5 cm downstream along the diffracted beam path from the first crystal (as defined by the iodine *K*-edge). The focus’ actual location was determined by measuring the beam size at multiple distances along the path of the beam and interpolating to the minimum beam width. This second crystal is then slowly rocked until a beam diffracted by the first crystal is also diffracted through the second crystal. This double-diffracted beam is now ‘parallel’ once again, but its horizontal dimension has been compressed fourfold. This beam is parallel in the sense that it is not converging or diverging any more than the original white beam, and also in the sense that it runs parallel to the original white beam, albeit shifted outboard by the action of the pair of crystals – analogous to a telescope in visible light (or, more precisely, one half of a porro prism binocular set).

A sample on a set of motorized stages with rotation was placed in this beam and a detector downstream of the sample. The sample stage included rotation adjustments (pitch and roll), as well as inboard/outboard location to align the CT stage with respect to the beam and detector. The center of rotation of the sample stage and the iodine *K*-edge were aligned with the center of the detector.

### 2.1. System energy calibration

Energy calibration of the beam is not essential for qualitative imaging, but, if quantitative analysis is required, the energy of X-rays at each point in the detector must be determined. A number of steps were required to calibrate the energy dispersion on the detector.

**Figure 4:**
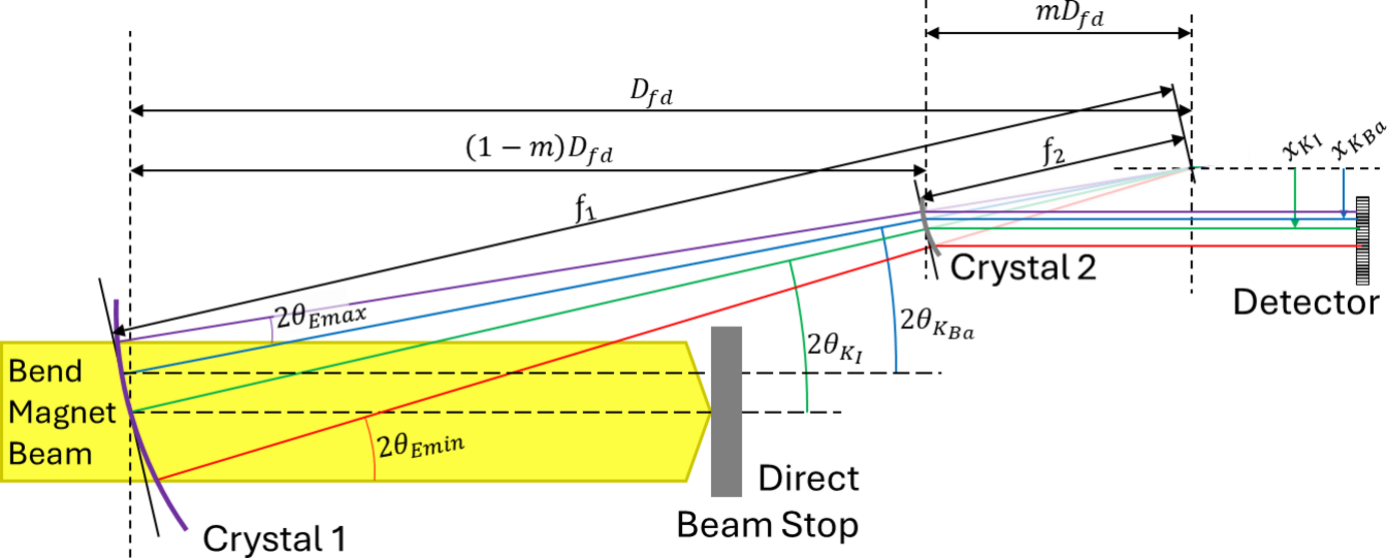
Compressor layout. A schematic layout of the two-crystal compressor. Crystal 1 has a focal length of *f*_1_ and crystal 2 has a demagnified focal length of *f*_2_ = *mf*_1_. Crystal 2 has the same orientation, uses the same reflection as crystal 1 and is located to diffract all the beam from crystal 1. This two-crystal geometry throws the diffracted beam parallel if the incident beam is parallel in the plane shown. The low- and high-energy rays are shown along with the two *K*-edge energy rays used for system energy calibration at the detector.

The calibration was based on measuring the locations of *K*-edges of two reference materials (iodine and barium) along with energy scale verification using the additional *K*-edges of caesium and gadolinium. The calibration process used Bragg’s law and the two reference *K*-edges (iodine at 33.169 keV and barium at 37.441 keV) to determine the detector location relative to the focal location, *D*_fd_, by using the pixel locations of the edges in the detector. The factor *m* is the magnification which is 1/4 in this case. The two *K*-edge locations, *x*_*K*I_ for iodine and *x*_*K*Ba_ for barium, were determined by taking the derivative of the normalized edge images and fitting the peak centers. A schematic of the geometry is given in Fig. 4 ▸. The locations then determine *mD*_fd_ using
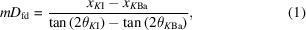
where θ_*K*I_ and θ_*K*Ba_ are the Bragg angles for the iodine and barium *K*-edge energies, respectively. The Bragg angles are found from Bragg’s law, the energies and the Si (3,1,1) *d* spacing, *d*_311_ (0.1637 nm).

Once the focus-to-detector distance is determined, the energy calibration, *E*_*i*_, for all detector pixels, *x*_*i*_, can be found by
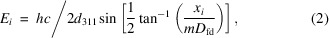
where *hc* = 1.24 nm keV. With this calibration, the caesium*K*-edge was found at an energy within about 10 eV of the tabulated value.

**Figure 5:**
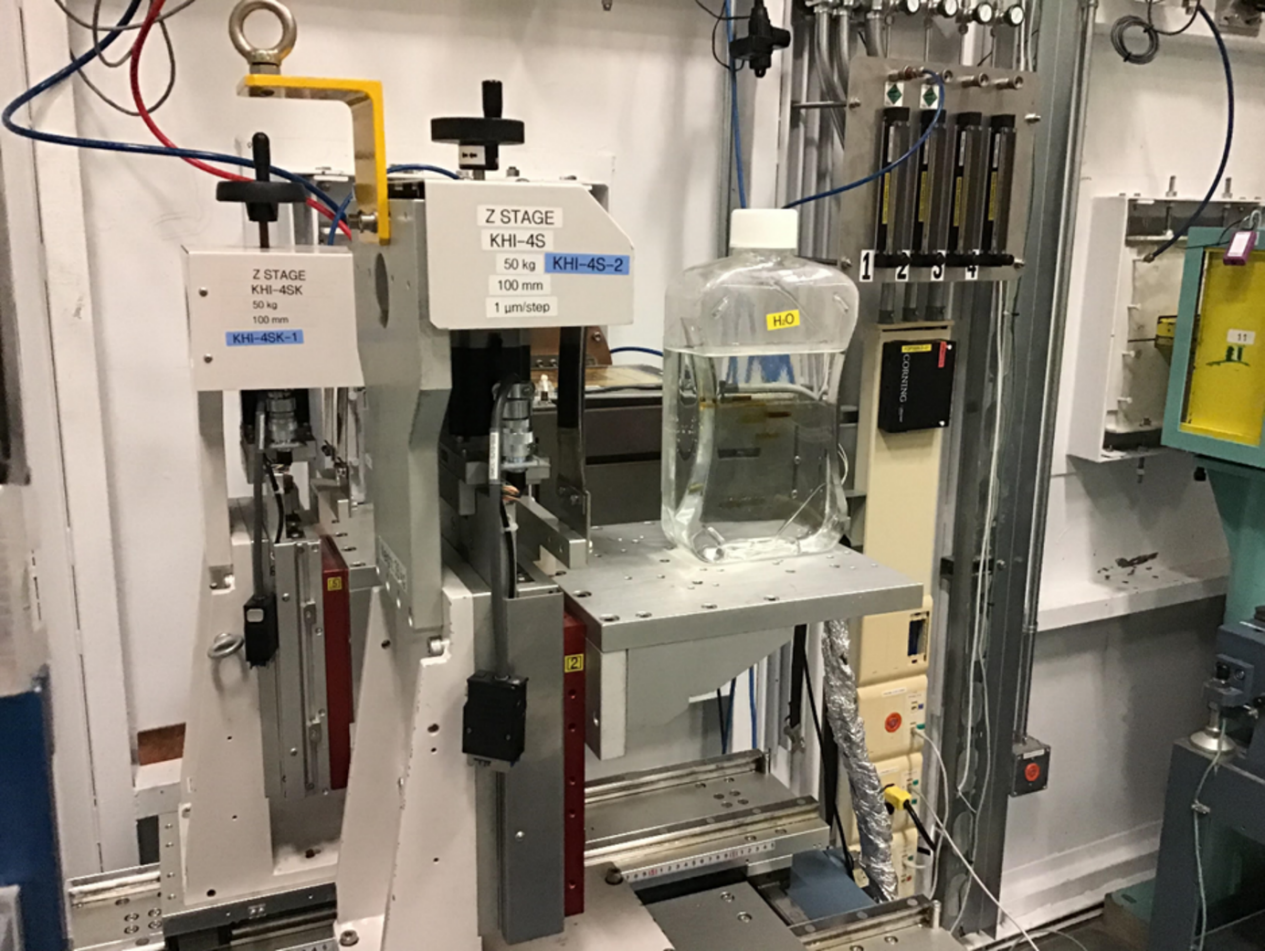
Tank of water used as an incident beam spectral flattening filter, upstream of the crystals.

A simple beam spectral flattening filter was employed, namely 61.7 mm of water, see Fig. 5 ▸. This filter reduced the flux of the lower X-ray energies to make the overall spectral flux more constant.

**Figure 6:**
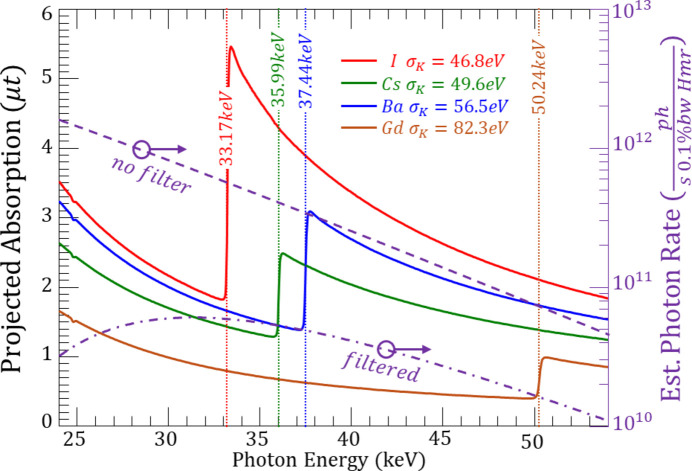
Measured *K*-edges of I, Cs, Ga and Gd based on the calibration using the I and Ba *K*-edges. Also shown is the measured *K*-edge widths for each element based on a Gaussian fit to the energy derivative of each edge. The violet dashed and dot–dashed lines with scale on the right indicate the calculated incident photon rate to the compressor without (dashed) and with the combined aluminium and water (dot–dashed) filters, showing the effect of spectrally flattening the imaging beam.

The *K*-edges used for energy calibration and energy scale validation are shown in Fig. 6 ▸. The additional *K*-edges of caesium and gadolinium were found to be within 10 eV of the tabulated values. A measure of the energy resolution is indicated in the legend of Fig. 6 ▸. The measured energy widths of the four elements are given and vary from 46.8 eV for iodine to 82.3 eV for gadolinium. It should be noted that the pixel-to-pixel average energy values vary from 24.8 eV at iodine to 54.9 eV at gadolinium due to the highly non-linear bahaviour of equation (2). Fig. 6 ▸ also shows the calculated effect of the 61.7 mm water and 1.1 mm aluminium spectral flattening filters which reduce the low- to higher-energy variation in the flux from a factor of 34 to 4.6 over the plotted energy range. The variation with the aluminium filter alone is a factor of 20, indicating the usefulness of the water filter to provide a more spectrally flat imaging beam.

### 2.2. Imaging procedure

Images were captured with a Hamamatsu CMOS flat-panel detector (Hamamatsu Photonics K.K., Solid State Division, Hamamatsu City, Japan; model C9252DK-14), placed downstream from the second crystal and sample stage (see Fig. 3 ▸). The beam was aligned with the top 1 cm section of the detector which has a 100 × 100 µm pixel size.

CT scans through 360° were performed and reconstructed on several samples of varying complexity. We include scans taken of a nylon screw and of an intact barley head to demonstrate proof-of-principle of CT with the compressed beam.

CT datasets were reconstructed using the filtered back-projection approach in IDL (Interactive Data Language, NV5 Geospatial, Broomfield, Co., USA) and MATLAB (MathWorks, Natick, Mass., USA). Each sinogram covers the full energy range of the beam within the image width, for a vertical axis of rotation. If a horizontal axis of rotation had been used in CT scans, each sinogram would be at a single energy with the imaging energy changing from slice to slice of the object.

## 3. Results

**Figure 7:**
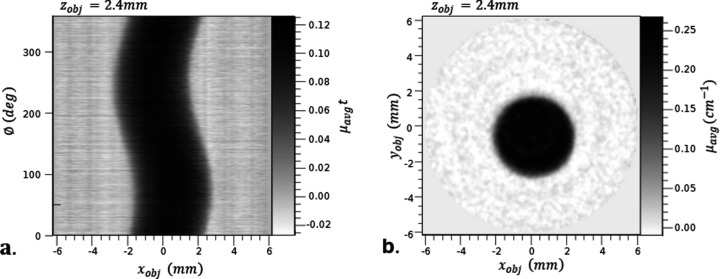
Sinogram (*a*) and axial view (*b*) of a CT scan of a nylon screw taken with the compressed beam produced by the parallel-beam compressor.

**Figure 8:**
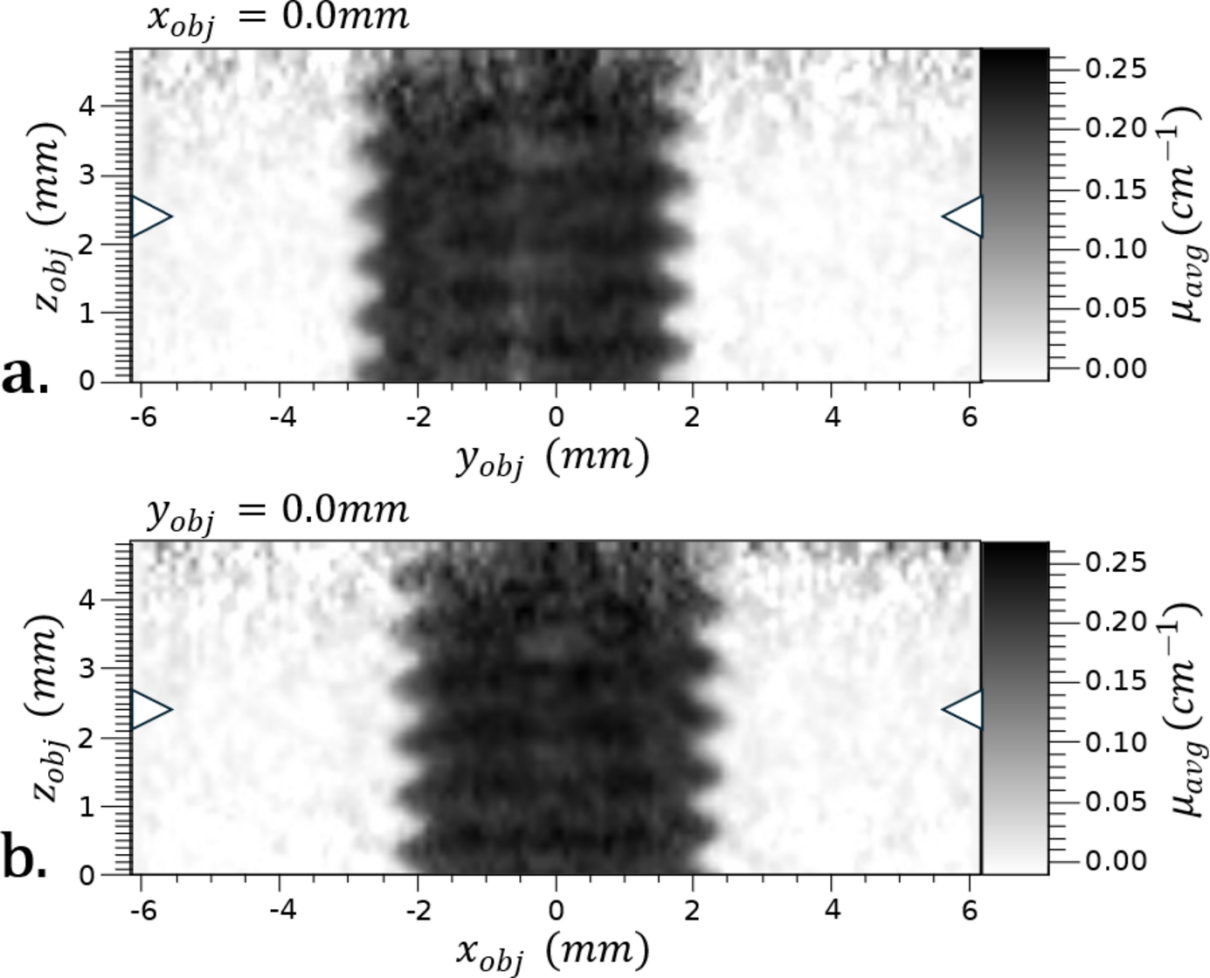
Orthogonal medial plane views of the CT of the nylon screw just shown. Taken with the compressed beam produced with the parallel-beam compressor. Left and right arrows identify the location of the images shown in Fig. 7 ▸.

We present proof-of-concept CT scans of a nylon screw, including the sinogram of a slice of the CT data, as well as axial and medial views of the reconstructed dataset (see Fig. 7 ▸ and Fig. 8 ▸). This demonstrates the feasibility of the compressor. The simplicity of the sample provides straightforward verification that the scans accurately represent the sample, in a qualitative manner.

**Figure 9:**
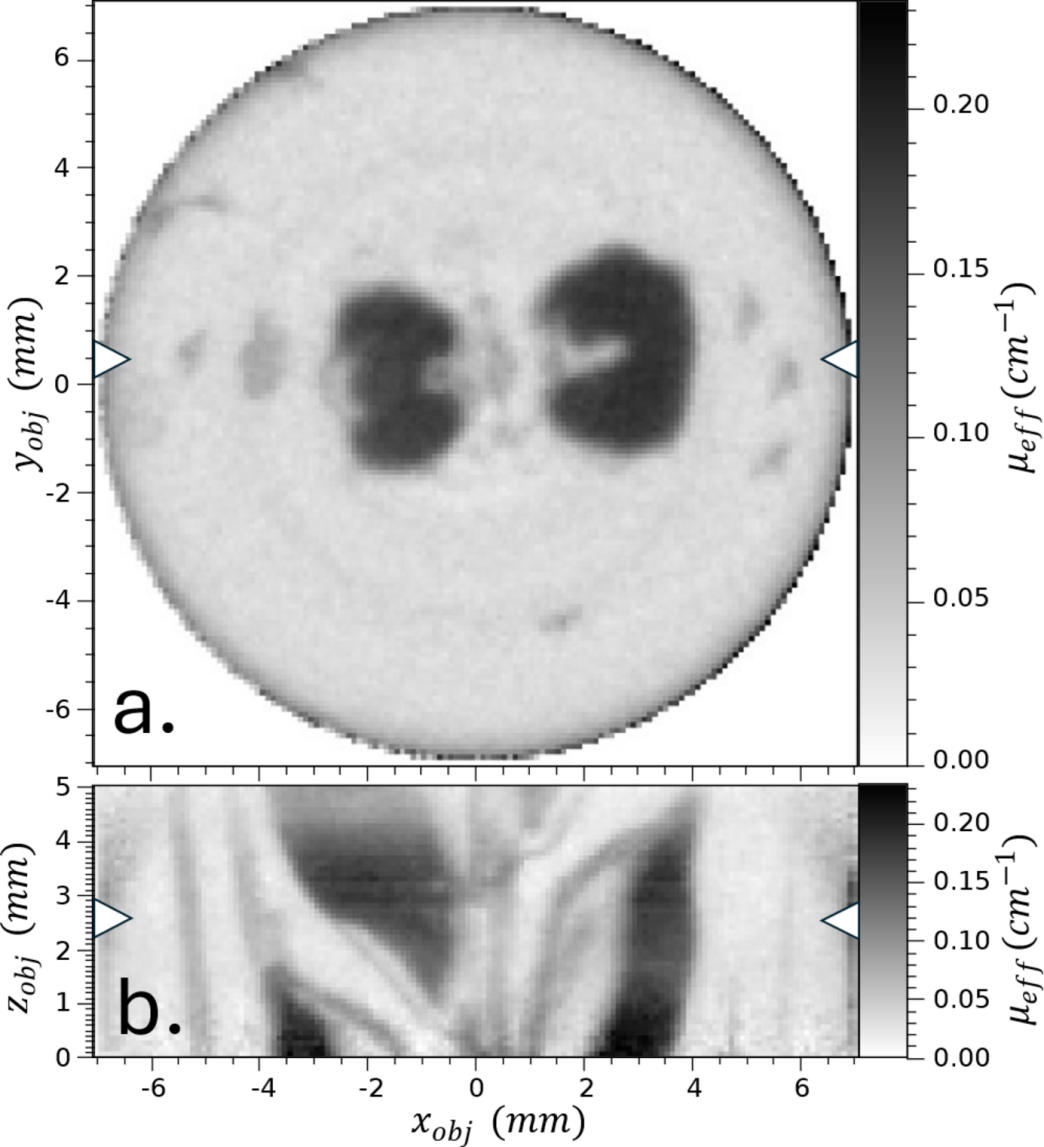
Axial (*a*) and transverse (*b*) views of a two-row barley head. The reconstructed volume was 14.2 mm × 14.2 mm × 5.1 mm along *x*, *y* and *z*, respectively. The arrows in each view indicate where the axial and transverse views intersect.

Additionally, we present CT scans of an intact head of barley grain (see Fig. 9 ▸). This demonstrates proof-of-principle with a more complex sample. We do not explore image refinement in this proof-of-principle research.

## 4. Discussion

The CT scans show implementation of the beam compressor for CT imaging of small samples. Resolution is quite limited for sample size, due to the detector chosen, but sufficient to establish proof-of-principle. There are several points of consideration concerning this compressor.

### 4.1. Spectral nature of the compressed beam

The energy range of the full spectrum of the imaging beam is determined by the range of angles the incoming beam makes with the crystal lattice planes. For the 4× compression demonstrated in this research, an energy range of 29.52 keV to 37.86 keV was produced. The incident beam was compressed from 56 mm to 14 mm.

For present applications, during a CT scan in the compressed beam with a vertical axis of rotation, a sample will be interrogated by a variety of energies as it rotates through the scan. Should one wish to avoid this situation, a horizontal axis of rotation may be implemented, such that each slice of the reconstructed CT dataset is taken at a single, known energy. A horizontal orientation of the object would require a rigid sample to avoid flexing during the rotation.

In the CT scans performed for this research paper, the spectral nature of the beam did not prove to present issues in data acquisition or reconstruction, but its effects on the resulting data should be carefully considered if conclusions based on the quantification of attenuation coefficients of voxels are to be made. This will be explored more fully in future papers.

### 4.2. Dose

The dose rate after the second compressor crystal was measured using an ionization chamber (IC Plus 150, XDS Oxford Ltd, Oxford, UK) and electrometer. The chamber accepted the full vertical size of the beam and used a 1.5 mm-wide slit to aperture the horizontal width. The slit and chamber were scanned across the width of the compressed beam, giving an average dose rate over the full vertical size of the beam (4.8 mm at 25 m) which was found to be 29.4 mGy s^−1^ at an energy of approximately 30.7 keV. Accounting for the vertical distribution of the beam, the dose rate varied from 46.2 mGy s^−1^ to 8.2 mGy s^−1^. The photon flux rate average was 4.32 × 10^10^ photons s^−1^ cm^−2^ and varied from 6.92 × 10^10^ photons s^−1^ cm^−2^ to 1.21 × 10^10^ photons s^−1^ cm^−2^.

The expected maximum dose rate based on a two-crystal lamella model (Etelaeniemi *et al.*, 1989 ▸; Erola *et al.*, 1990 ▸) was approximately 140 mGy s^−1^, also at 220 mA. The discrepancy is mostly due to not achieving uniform bending of the two crystals and thus a mismatch in the relative Bragg angle of the lattice planes across the crystals.

For reference, the measured and predicted peak dose rate of the Si (2,2,0) double-crystal monochromator in the beamline was 24 mGy s^−1^ at the 33 keV central energy of the compressor, also at 220 mA ring current.

### 4.3. Advantages

Compared with the monochromatic beam of the BM beamline, there are several potential advantages to the beam compressor, and a few potential disadvantages, depending on which imaging techniques the beam compressor is used with.

The major limitation of BM beamlines in modern synchrotrons is in their intensity, as compared with insertion-device (‘wiggler’) beamlines. The greatly increased intensity of insertion-device beamlines makes them very appealing; however, that intensity comes with some challenges, including excessive heat on beamline optics and dose delivered to samples, making stability and damage control quite unforgiving. The introduction of a horizontal compressor, downstream from standard beamline optics, in a BM beamline allows a flexible, user-customizable increase in intensity.

The compressor increases intensity in two ways: firstly, it horizontally compresses the beam, funneling photons from unused portions of the beam into the field of view, and, secondly, it increases the reflectivity width/energy bandwidth due to the curvature of the crystal (Muehleman *et al.*, 2009 ▸; Suortti & Schulze, 1995 ▸; Sparks *et al.*, 1980 ▸; Rhoades, 2015 ▸; Erola *et al.*, 1990 ▸).

The beam produced by the compressor is spatially monochromatic, with the energy of monochromatization changing across the beam (and therefore the detector and images) from inboard to outboard, or left to right. The energy spectrum is well ordered and known, having been established by Bragg’s law and imaging of *K*-edges as shown in equation (2).

Lastly, compression of the beam reduces the horizontal aspect of the effective source size and increases the effective horizontal source divergence, according to the optical invariant principle.

To summarize, the advantages are listed below:

(i) Customizable, increased monochromatic beam intensity at the sample.

(ii) No increase in intensity at beamline optics, *e.g.* windows, collimators, filters, *etc*.

(iii) Shorter exposure times enabling quicker scans and higher throughput of samples.

(iv) Reduced pressure on insertion-device beamlines by improving the practicality of BM beamlines for high-resolution imaging.

(v) Highly regulated spectrum of energies across the direction of compression.

(vi) The compression will result in a smaller effective horizontal source size, at the expense of a larger effective horizontal source divergence.

### 4.4. Disadvantages

There are some disadvantages to using the beam compressor. Firstly, setup of the system (particularly the second crystal) is quite time-consuming. In order for the alignment of the second crystal to meet the diffraction conditions established by the first crystal, a very high level of precision is required and there is no beam diffracted at all until those conditions are met (Rhoades *et al.*, 2015 ▸; Zhang *et al.*, 2007 ▸; Martinson *et al.*, 2017 ▸). The second crystal must be aligned with the diffracted beam spatially, along the axis of the diffracted beam (in our case, about 13.5° off the typical beam path), at the correct distance from the focus based on the actual curvature of the crystals, and the Bragg angle adjusted to within a single reflectivity width of the first crystal. This alignment process requires several steps and increases setup time considerably. This setup time could be reduced by mounting the pair of crystals to a single plate such that they are aligned permanently. This apparatus of the crystal pair might then be placed in the beam and aligned therewith, eliminating the need to align the second crystal relative to the first each time the compressor is used. Development and testing of such a mounting system is beyond the scope of this research paper, which is proof-of-principle in nature.

It should be noted that any imperfections in the curvature of either of the crystals or slight misalignments between portions of the two crystals will reduce the intensity of the compressed beam at the point of misalignment (Martinson, 2017 ▸). This could both reduce the overall intensity and compromise the uniformity of the imaging beam which we observed in these experiments. This issue was explored with the beam expander (Martinson *et al.*, 2014 ▸) with its large beam area on the second crystal.

Secondly, there is a potential disadvantage for some types of imaging. As explained previously, the compressed beam is spectral in nature in the compressed dimension. Energies are well ordered and known, but this spectral nature may complicate the analysis of data for some applications. For CT of a sample using a vertical axis of rotation, the sample is imaged by a variety of different energies. For CT of a sample using a horizontal axis of rotation, each slice within the dataset is taken at a different beam energy. This effect of the beam compression offers some potential for further information to be extracted, which we intend to explore in the future.

Lastly, while the compressor increases the intensity of the beam relative to the non-compressed monochromatic beam of a BM beamline, it does not increase the intensity of the beam relative to the unfiltered white beam. For experiments that do not require monochromatization, the compressor does not provide an increase in beam intensity.

These disadvantages are summarized below:

(i) Setup times are greatly increased.

(ii) The beam is monochromated at different energies, spatially, across the direction of compression.

(iii) There is no increase in intensity over the white or filtered white beam.

(iv) As noted earlier, the horizontal beam compression will result in a larger effective horizontal source divergence with a smaller effective horizontal source size.

### 4.5. Potential applications

The beam compressor makes it possible to increase the capabilities of a BM beamline using only two inexpensive, thin, bent silicon crystals. This may provide value to researchers performing high-resolution CT. Beam intensity is very important to high-resolution CT, because increasing resolution while maintaining good signal-to-noise ratios increases scan times drastically in CT (Ford *et al.*, 2002 ▸). Refinement of the double-crystal setup could increase the intensity over the results presented in this feasibility study. As noted earlier, if the double-crystal compressor were to be used repeatedly, a setup wherein both crystals are mounted to a single plate to maintain relative crystal alignment would be useful.

The compressed beam will contain a range of energies which could be used for *K*-edge subtraction CT and/or spectral CT for material characterization.

## 5. Conclusion

The ability to horizontally compress, rather than collimate, the beam provides a more efficient and convenient use of BM beamlines for some imaging applications. We present a horizontal beam compressor utilizing bent Laue crystals to provide a fourfold compression and increase beam intensity relative to traditional monochromatic beams, while obtaining a useful aspect ratio. Implementation of a beam compressor allows this beam aspect to be adjusted to much more practical ratios for small samples, by utilizing, rather than discarding, the photons outside the useful portion of the beam.
